# Prevalence of Needlestick Injury and Its Potential Risk among Veterinarians in Nigeria

**DOI:** 10.1155/2016/7639598

**Published:** 2016-10-17

**Authors:** Philip Paul Mshelbwala, J. Scott Weese, Jibrin Manu Idris

**Affiliations:** ^1^Department of Veterinary Medicine, Faculty of Veterinary Medicine, University of Abuja, Abuja, Nigeria; ^2^Department of Pathobiology, Ontario Veterinary College, University of Guelph, Guelph, ON, Canada; ^3^African Field Epidemiology Network, Abuja, Nigeria

## Abstract

A cross sectional study using multistage sampling method by means of structured interviewer administered questionnaire was designed to estimate the rate of occurrence of needlestick injuries among veterinarians involved in clinical practice and to evaluate needle handling practices and risk factors. The study was carried out during the months of August–November 2015. Out of the 215 veterinarians that participated in the survey, 171 (79.5%) reported to have suffered needlestick injuries (NSIs). In the multivariable model, only male sex (OR 2.8, 95% CI 1.4–6.0, and *P* = 0.006) and working with poultry daily (OR 2.4, 95% CI 1.1–6.2, and *P* = 0.036) were significantly associated with NSI. Most (111, 64.9%) veterinarians had discomfort including pain, headache, fever, worry, and local numbness from NSIs; however, none was hospitalised. Only 1 (0.6%) had lost time at work. The approach to needlestick injury avoidance was poor and most (98.8%) NSIs were not reported. The findings of this research call for comprehensive health and injection safety programs for veterinarians involved in clinical practice.

## 1. Introduction

Accidental puncture of the skin by a needle, otherwise known as “needlestick” or “needlestick injury” (NSI), can occur before, during, and after a procedure, before, during, and after improper needle disposal (e.g., leaving needles in a laboratory coat with subsequent needlestick injury to laundry worker [1]), or at any other time in the process where a needle is handled. Needlestick injuries pose risks from injection of contents, exposure to pathogens, and physical trauma. Infectious disease risks include exposure to blood-borne pathogens, organisms from the animal's or person's skin (*Staphylococcus* spp.) or from fine-needle aspirates (*Pasteurella* spp.,* Staphylococcus* spp.,* Streptococcus* spp., and* Blastomyces*), or modified live vaccines [1]. Severe laceration such as an NSI occurring during animal movement during injection or blood collection can be significant. Even limited trauma can result in potentially serious consequences in some locations, such as injuries associated with joints, nerves, tendons, and bone. Exposure to medications and vaccines also poses potential risk of reaction to the medication, including typical drug effects (e.g., sedatives), allergic effects (e.g., penicillin allergy), toxic effects (e.g., tilmicosin exposure), and idiosyncratic effects.

In human medicine, considerable time, effort, and resources have been put in place to reduce the incidence of NSIs largely driven by the infection of healthcare workers (HCWs) with infectious agents such as hepatitis B virus, hepatitis C virus, and human immunodeficiency virus (HIV) [2]. However, in veterinary medicine, a similar proactive approach towards NSIs is lacking; this is likely due to poorly developed culture of concern regarding occupational health and safety in the profession and because serious blood-borne zoonotic pathogens of domestic animals are not recognised as important problems in most regions. There are over five thousand registered veterinarians in Nigeria [3]. Needlestick injuries are considered to be very common types of injuries among healthcare workers; however, the quality of available data is variable and it is believed that there is significant underreporting [4]. The incidence of NSIs might be higher in veterinary medicine given the potential laxity in sharps handling practices because of fewer identified blood-borne pathogens risks. There have been few studies of the incidence of NSIs in veterinary medicine when compared with human medicine, but available data indicate concerningly high rates [1]. For example, in a study conducted among female veterinarians, 64% reported one or more NSIs in their career, with vaccines accounting for 50% of the incidents [5]. In study conducted by Ansa et al. [6], in three health institutions from South West Nigeria, they observed that basic protective equipment supplies were grossly inadequate in all the health institutions and safety practices were not adhered to; all these could increase the risk of contracting blood-borne infections. About three million healthcare workers are estimated to experience percutaneous exposure to blood-borne pathogens annually [7]. The majority of the cases occur in developing countries in Africa, probably due to poor injection safety practices and lack of basic supplies. The prevalence of NSI in Africa among healthcare workers varies from 31% to 68%; it is 30.9% in Southern Ethiopia, 52.9% in Tanzania, 67.9% in Alexandria in Egypt, and 68% among gynaecologists in Nigeria [8]. However, in Nigeria and other developing countries of Africa, corresponding data are lacking among veterinarians. Understanding NSI rates and risk factors is important for identifying education and intervention needs to reduce the incidence and impact of NSIs among veterinary personnel. This study was designed to estimate the rate of occurrence of NSIs among veterinarians involved in clinical practice in Nigeria and to evaluate needle handling practices and identify factors associated with NSIs. Inferences drawn from this study will be used in the development of effective risk reduction strategies.

## 2. Materials and Method

A cross sectional study using multistage purposive sampling method was performed by means of self-administered structured interviewer questionnaire (available in the Supplementary Material online at http://dx.doi.org/10.1155/2016/7639598). The questionnaire was developed and pretested among 32 veterinarians involved in clinical practice in two veterinary teaching hospitals in Nigeria. The questionnaire was thereafter administered to veterinarians who were willing to participate in the study across six veterinary teaching hospitals in the country and during the 52nd annual conference of the Nigerian Veterinary Medical Association (NVMA), Rivers States University of Science and Technology, Diobu area of Port Harcourt, Rivers State, Nigeria, 16–20 November 2015. The theme was “Providing Holistic Solutions to the Staggering Nigerian Economy: Challenges and Opportunities for Veterinarians.” Veterinarians who attended the conference came from all the states of the federation and from different universities. The questionnaire was administered to all volunteers that were involved in clinical practice. Respondents were briefed on the purpose of the study and their consent was sought. The questionnaire covered various aspects of demographics, practice type, qualification, years in practice, NSIs, and injury reporting. Multiple choice and open-ended questions were included. Retrospective incidence data were collected by asking questions related to activity over past week, month, and year and over career. Respondents were at liberty to skip questions they did not want to answer. Data were analysed using Statistical Package For Social Science (SPSS) Version 17. Univariable logistic regression models were constructed to estimate the association between having experienced an NSI and a variety of independent variables. Continuous independent variables were assessed to determine whether there was a linear relationship between the variable and the log odds of the outcome, with continuous data transformed if required. All significant variables based on a liberal *P* value (i.e., *P* < 0.25) in the univariable analysis were considered for inclusion in the multivariable model. The multivariable model was fitted using a backwards stepwise approach to create a main effects model using a significance level of *α* = 0.10 while retaining confounding variables regardless of statistical significance. Confounding was assessed by examining the change in the coefficients for the remaining significant variables after removal of the potentially confounding variable. If the coefficient for one of these variables changed more than 20%, the removed variable was considered a confounder and was retained in the model. A *P* value of < 0.05 was considered significant. Model fit was assessed after completion of the final multivariable model.

## 3. Results

Two hundred and fifteen veterinarians completed the questionnaire; 159 (74%) were male and 65 (26%) female. This represented 49% (197/404) from the conference attendees and 18 from the teaching hospitals. One hundred and twenty (55.8%) had Doctor of Veterinary Medicine (DVM) degree, 77 (35.8%) had M.S. degree, and 18 (8.4%) had a Ph.D. degree. Needlestick injuries were very common, with 171 (79.5%) reporting at least one NSI during their career. Most veterinarians had experienced numerous NSIs (Table 1). A total of 216 NSIs were estimated to have occurred, with more cases among those who handled poultry and dogs. Univariable data are reported in Table 2. In the multivariable model, only male sex (OR 2.8, 95% CI 1.4–6.0, and *P* = 0.006) and working with poultry daily (OR 2.4, 95% CI 1.1–6.2, and *P* = 0.036) were significant. Model fit testing indicated that the data fit the model (*P* = 0.80).

Veterinarians were carrying out various tasks during the last NSIs, with recapping of needle (50, 29.2%) being the main reason for NSIs (Table 3).

Various reasons were implicated by veterinarians as causative factors for NSIs (Table 4) with poor restraint as the major cause (42.7%). A variety of substances were present in syringes associated with NSIs, with antibiotics (57.9%), killed vaccines (15.2%), and modified live vaccines (3.5%) being the most common (Table 5). Most (111, 64.9%) respondents had experienced discomfort from the NSI, including pain, headache, fever, worry, and local numbness; however, none was hospitalised. Only 2 (1.2%) sought medical care. The majority (148, 86.5%) applied antiseptic at the site, while 14 (8.2%) did nothing. Information on needle handling practices indicated that most veterinarians do not have sharp containers in their clinics. The majority of respondents (128, 59.5%) reported not having access to a sharps container in their clinic, with only 66 (30.7%) responding to a sharps container and 21 (9.8%) declining to respond. Most respondents place used syringes in sharp container after recapping (Table 6). The majority of respondents (195, 90.7%) knew NSIs can transmit diseases and 171 (79.5%) indicated they needed injection safety training.

## 4. Discussion

The high incidence of NSIs was concerning but consistent with previous veterinary studies [4, 9, 10] as well as a study of gynaecologists in Nigeria [11]. This high occurrence of NSIs raises concerns because of the potential for adverse effects and the deficiencies in preventive and reporting practices that potentiate the risk of NSIs and hamper understanding of the problem.

Various factors were associated with increased odds of NSI in the univariable analysis, but only being male and working with poultry daily remained significant in the final multivariable model. The increased odds of NSI among male veterinarians is interesting and should prompt consideration of why this occurs. It may be due to behavioural differences and less care when handling sharps; however, those details were not possible to investigate in this study.

The association with poultry practice, but not other clinical practices, was interesting and has not been previously reported. This could be associated with a larger number of NSI opportunities, through handling and injection of larger numbers of animals, compared with work on other species. This finding indicates a need to target poultry practitioners for education campaigns regarding sharps handling practices and NSI avoidance.

A variety of practices are recommended to reduce the incidence of NSIs. These include not recapping needles by hand, disposing of needles directly into an approved sharps container, never leaving uncapped needles on a surface, never leaving needles in laundry, and ensuring good restraint [12]. Poor compliance with these recommendations was identified in this study, something that has been identified in previous surveys and an observational study in veterinary medicine [9, 13]. Recapping was the most common event associated with NSI, something that is avoidable but common in veterinary medicine; this agrees with the report of Anderson and Weese [9]. Manual recapping is a leading cause of NSIs through missing the cap and puncturing a finger or driving the needle through the side of the cap and into a finger. This is a modifiable activity and reducing recapping would probably have a profound impact on NSI rates. Poor restraint was also commonly implicated as a cause of NSI. Poor restraint can create risk of self-puncture when injecting a struggling animal or when the veterinarian is distracted by the poorly restrained animal. Improvements in patient handling along with associated aspects such as avoiding rushing and ensuring adequate restraint prior to injection can probably impact NSI risk. Stress and fatigue were other reported factors, both of which could lead to decreased attention, decreased care, and rushing to inject in the absence of proper restraint. In human medicine, there is increasing use of “safety engineered devices” such as retracting needles or capping systems to reduce any chance of NSI [14]. The added cost and decreased attention to NSIs in veterinary medicine likely account for limited use of these approaches, particularly in developing countries.

Poor compliance is a common problem with many infection control practices, including needle handling. Various reasons can be present, including lack of education, lack of motivation, inadequate supervision, inadequate time, and a lack of supplies or infrastructure. All these could play a role in the results of this study. Only 56% of participants reported placing their used syringes in a sharps container after recapping, indicating a lack of education or motivation about the risks of recapping. The lack of ready access to proper sharps disposal containers, something that would preclude safe needle handling, was another problem, with 20.9% of veterinarians not having a sharps disposal container in the clinic. This lack of basic supplies precludes safe needle handling; this is similar to the report of Ansa et al. [6] who carried out a study in three health institutions from South West Nigeria. They observed that basic protective equipment supplies were grossly inadequate in all the health institutions and safety practices were not adhered to, factors that could increase the risk of contracting blood-borne infections. Some clinicians (30.7%) placed their used syringe and needle in their laboratory coat temporally following use, a high risk practice as either the veterinarian or laundry personnel is likely to sustain an NSI during wearing of the coat or laundering, exposing both the clinician and other personnel to risk.

Education is an important aspect of infection control and occupational health. While needles are handled very regularly, knowledge about optimal handling practices may be variable. This perception may indicate a lack of understanding given the widespread inadequacy. Veterinarians were exposed to a variety of substances (Table 5) from NSIs. Antibiotics were the most common. The high rates of exposure to antibiotics is of great concern due to the potential problems from the direct effects of drugs and allergic reactions [15].

The very low reporting rate of NSIs (1.2%) was perhaps unsurprising but nonetheless concerning. Reporting rates were slightly higher but still shockingly low (9.2%) in a study of gynaecologists in Nigeria [11], highlighting a gap in education about the importance of NSIs and the need for reporting. Reporting of NSIs is important to ensure that any indicated prophylactic care is provided and to collect accurate NSI data. While medical care was rarely sought, the majority of participants reported applying an antiseptic following the injury. However, while antiseptics may be useful to reduce the risk of infection, they do not eliminate that risk or address other relevant issues such as local inflammation and systemic reactions.

While NSI rates among veterinarians are high, limited information is available about the incidence of infectious diseases associated with NSIs. While anecdotal, two respondents reported to have known veterinarians who came down with brucellosis and trypanosomiasis as a result of NSIs, while 9 (0.04%) had themselves developed conjunctivitis following exposure to Newcastle Disease Vaccine. Considering the limited reporting of NSIs and the lack of mandatory reporting system for NSI associated infections, unreported infections almost certainly occur. There was no association between the incidence of NSIs among veterinarians in private practice and those working with the veterinary teaching hospitals.

This study has demonstrated that NSIs are very common among veterinarians involved in clinical practice in Nigeria and that potentially unsafe practices are widespread. The frequency of injuries and the poor approach to needlestick avoidance call for comprehensive health and injection safety programs for veterinarians involved in clinical practice. The major limitations of this study are lack of data about wearing gloves, the localization of NSI, and the occurrence of observable NSI through regular visits to various practice fronts.

## Supplementary Material

Self-administered structured interviewer questionnaire which is used to perform cross sectional study using multistage purposive sampling method.

## Figures and Tables

**Table 1:** Number of needlestick injuries reported by veterinarians.

Number of needlestick injuries	Number of veterinarians
1–4	51 (29.8%)
5–8	34 (19.9%)
8–10	50 (29.2%)
>10	36 (21.1%)

**Table 2:** Univariable logistic regression results for variables that entered the model based on a *P* ≤ 0.25.

Variable	Referent	Category	Odds ratio (95% CI)	*P* value
Sex	Female	Male	3.1 (1.6–6.3)	0.002

Frequency of treating dogs	Never	Daily	8.3 (2.3–35.8)	0.001
		Weekly	4.4 (1.2–16.4)	0.022
		Greater than weekly	3.2 (1.0–10.4)	0.049

Frequency of treating horses	Never	Greater than weekly	2.6 (1.2–5.5)	0.01

Frequency of treating poultry	Never	Daily	4.5 (1.2–16.9)	0.03

Frequency of treating goats	Never	Daily	3.3 (1.1–10.8)	0.036
		Weekly	5.7 (1.8–20.0)	0.002

Frequency of treating sheep	Never	Daily	4.1 (1.2–16.2)	0.02
		Weekly	4.6 (1.5–16.0)	0.007

Frequency of treating cattle	Never	Daily	5.5 (1.3–37.8)	0.019
		Weekly	4.1 (1.4–12.9)	0.008
		Greater than weekly	3.1 (1.4–7.1)	0.006

Temporary placement of syringes in laboratory coat following use	Yes	No	2.1 (0.88–5.3)	0.11

Work type	Government or university	Private practice	1.7 (0.75–4.6)	0.023

**Table 3:** Activity carried out during the last needlestick injury (*n* = 171).

Activity	
Withdrawing drug	14 (8.2%)
Collecting blood sample	14 (8.2%)
Manipulating needle in patient	37 (21.6%)
Handling garbage	5 (5.9%)
Handling uncooperative patient	26 (15.2%)
Recapping of needle	50 (29.2%)
Taking off cap	3 (1.8%)
Other	6 (3.5%)
No response	1 (0.6)

**Table 4:** Reasons for needlestick injury (*n* = 171).

Factor	
Long working hours	12 (7.0%)
Inappropriate environment	12 (7.0%)
Stress	24 (14.0%)
Inappropriate training	6 (3.5%)
Poor lightning	22 (12.9)
Poor restraint	73 (42.7%)
Other	11 (6.4%)
No response	11 (6.4%)

**Table 5:** What was contained in the syringe (*n* = 171)?

Content	
Killed vaccine	26 (15.2%)
Live vaccine	6 (3.5%)
Antibiotic	99 (57%)
Multivitamin and other	32 (18.8%)

**Table 6:** What do you do with used syringe and needle?

Place in sharp container after recapping	121 (56%)
Sometime place in sharp container	14 (6.5%)
Do not have sharp container	45 (20.9%)
Always place in sharp container without recapping	17 (7.9%)

## References

[1] Weese J. S., Jack D. C. (2008). Needlestick injuries in veterinary medicine. *Canadian Veterinary Journal*.

[2] Haiduven D. J., Simpkins S. M., Phillips E. S., Stevens D. A. (1999). A survey of percutaneous/mucocutaneous injury reporting in a public teaching hospital. *Journal of Hospital Infection*.

[3] Nigerian Veterinary Medical Association, 2016, http://nvma.org.ng/

[4] Elder A., Paterson C. (2006). Sharps injuries in UK health care: a review of injury rates, viral transmission and potential efficacy of safety devices. *Occupational Medicine*.

[5] Wilkins J. R., Bowman M. E. (1997). Needlestick injuries among female veterinarians: frequency, syringe contents and side-effects. *Occupational Medicine*.

[6] Ansa V. O., Udoma E. J., Umoh M. S., Anah M. U. (2002). Occupational risk of infection by human immunodeficiency and hepatitis B viruses among health workers in South-eastern Nigeria. *East African Medical Journal*.

[7] WHO (2015). *Secretariat of the Safe Injection Global Network. Health Care Worker Safety*.

[8] Amira C. O., Awobusuyi J. O. (2014). Needle-stick injury among health care workers in hemodialysis units in Nigeria: a multi-center study. *International Journal of Occupational and Environmental Medicine*.

[9] Anderson M. E. C., Weese J. S. (2015). Video observation of sharps handling and infection control practices during routine companion animal appointments. *BMC Veterinary Research*.

[10] Mansour A. M. (1990). Which physicians are at high risk for needlestick injuries?. *American Journal of Infection Control*.

[11] Efetie E. R., Salami H. A. (2009). Prevalence of, and attitude towards, needle-stick injuries by Nigerian gynaecological surgeons. *Nigerian Journal of Clinical Practice*.

[12] Castella A., Vallino A., Argentero P. A., Zotti C. M. (2003). Preventability of percutaneous injuries in healthcare workers: a year-long survey in Italy. *Journal of Hospital Infection*.

[13] Cullen B. L., Genasi F., Symington I. (2006). Potential for reported needlestick injury prevention among healthcare workers through safety device usage and improvement of guideline adherence: expert panel assessment. *Journal of Hospital Infection*.

[14] Trim J. C., Elliott T. S. J. (2003). A review of sharps injuries and preventative strategies. *Journal of Hospital Infection*.

[15] Georgios C. (2009). Adverse outcome of using tilmicosin in a lamb with multiple ventricular septal defects. *Canadian Veterinary Journal*.

