# Supplementary material for: Prevalence of Needlestick Injury and Its Potential Risk among Veterinarians in Nigeria
Source: Vet Med Int. 2016 Oct 17;2016:7639598. doi: 10.1155/2016/7639598 (PMC5086500; doi:10.1155/2016/7639598)
Supplement: Supplementary file 1 — Self-administered structured interviewer questionnaire which is used to perform cross sectional study using multistage purposive sampling method. [file 7639598.f1.docx]

**PREVALENCE OF NEEDLESTICK INJURY AND ITS POTENTIAL RISK AMONG VETERINARIANS IN NIGERIA**

**Research Questionnaire**

**Consent from respondent**: We are from the Department of Veterinary Medicine Faculty of Veterinary Medicine University of Abuja. We are conducting a study on needlestick injury and its potential risk among Veterinarians in Nigeria

We are requesting your permission to give us the following information which will help in achieving the aim of the study. All information will be confidential and used for the purpose of this study only. Your participation is optional

Thank you.

Questionnaire Number------------------------- Date -------------------------------

**Demographic Information**

1. Sex Male [ ] Female [ ]
2. Highest qualification, DVM [ ] Masters [ ] PhD [ ]
3. Affilation, State and Fed Ministry [ ] University [ ] Reseach Institute [ ] Private Vet [ ] other specify …………………..

**Clinical Practices History**

1. How long have you been in Veterinary clinical practice? < 1 yr [ ] 1- 2 yrs [ ] 3- 5 yrs [ ] 6 – 10 yrs [ ] >10 yrs [ ]
2. How often do you treat these species of animals;

Dog: Daily [ ] Weekly [ ] Greater than weekly [ ] Never [ ]

Cat: Daily [ ]Weekly [ ] Greater than weekly [ ] Never [ ]

Horse: Daily [ ] Weekly [ ] Greater than weekly [ ] Never [ ]

Poultry: Daily [ ] Weekly [ ] Greater than weekly [ ] Never [ ]

Goat: Daily [ ] Weekly [ ] Greater than weekly [ ] Never [ ]

Sheep: Daily [ ] Weekly [ ] Greater than weekly [ ] Never [ ]

Cattle: Daily [ ] Weekly [ ] Greater than weekly [ ] Never [ ]

Pig: Daily [ ] Weekly [ ] Greater than weekly [ ] Never [ ]

1. Where do you carry out your practice? Clinic based practice [ ] Ambulatory [ ] Farm based practice [ ] All of the above [ ]
2. Do you have an attendant responsible for restraining animals in the clinic? Yes [ ] No [ ]
3. How many hours per week do you practice?---------

**Needle Injury Related History**

1. Have you ever accidentally inoculated yourself or suffered an accidental needle stick? Yes [ ] No [ ]
2. If yes to question 9 above, how often did this occur ? Daily [ ] weekly [ ] monthly [ ] yearly [ ] greater than yearl [ ]
3. If yes to questions 9 above, estimate the number of Needlestick injuries you had in your career ……….
4. If yes to 9 above …When was your last needlestick? Past 24 hour [ ] Past week [ ]

Past month[ ] Past 6 months[ ] Past year [ ] Greater than one year ago [ ]

1. Which activity were you carrying out during the last needle stick injury? Withdrawing drug from the bottle [ ] Collecting blood sample [ ] Manipulating needle in patient [ ] Handling garbage [ ] Surgery or suturing [ ] Handling uncooperative patient [ ] Recapping of capping [ ] Taking off cap [ ] others specify………
2. What may be the reason for needlestick ? Long working hours [ ] In-appropriate [ ] environment [ ] Stress [ ] In-appropriate training [ ] Poor lighting [ ] Poor restrain [ ] Others (specify)---------------
3. Did you experience any discomfort following your last needlestick? Yes [ ] No [ ]
4. If yes to 15 above , which of these did your experience ? Pain [ ] Headache [ ] Fever [ ] Worry [ ] Local Numbness [ ] Others………
5. Have you ever been hospitalized as a result of needlestick injury? Yes [ ] No [ ]
6. If yes to 17 above, indicate where and how long you were hospitalised ………………………………………………………………………………..
7. Have your ever missed work following a needlestick injury? Yes [ ] No [ ]

**Injected Safety Practices Related to Needlestick**

1. What did you do following the needlestick? Report to the clinic [ ] Did nothing [ ] Used antiseptic to clean [ ]
2. If your last needlestick involved a needle and syringe, which of these was contained in the syringe during the procedure? Killed vaccine [ ] Live vaccine [ ] antibiotic [ ] euthanasia agent [ ] anthelmintic [ ] Steroid [ ]
3. Have you had a needle stick injury following recapping - Yes [ ] No [ ]
4. Have you had any training on safe injection practices ? Yes [ ] No [ ]
5. What do you do with used syringes and needles? Always place in Sharp container without recapping [ ] Place in sharp container following recapping [ ] Sometimes place it in sharp container [ ] Do not have sharp container in the clinic [ ]
6. Do you temporary place used syringe in your laboratory court following usage, Yes [ ] No [ ]
7. Do you have a safety box for disposing used syringe following injection?Yes [ ] No[ ]
8. Are preventive facilities like trays/syringe containers, sharp disposal containers and antiseptic solution for needle stick injuries available in your clinic? Yes [ ] No [ ]

**Knowledge on Injection Safety**

1. In your view, which of these practices may carry high degree of risk of needle stick injury? (check all that apply) Recapping needle [ ] Transferring fluid into another container [ ] Withdrawing a needle from a patient [ ] Injecting animals [ ] Other source ……….
2. Do you know needle stick injury can transmit some diseases caused by viruses, bacteria, fungi and other micro-organism? Yes [ ] No [ ]
3. If yes to question 29 above, list any of the diseases ………………………..
4. Would you like to take part in a needlestick and sharp injury safety training? Yes [ ] No [ ]
